# Non-febrile COVID-19 patients were common and often became critically ill: a retrospective multicenter cohort study

**DOI:** 10.1186/s13054-020-03037-8

**Published:** 2020-06-08

**Authors:** Yichen Li, Na Jiao, Lixin Zhu, Sijing Cheng, Ruixin Zhu, Ping Lan

**Affiliations:** 1 The Sixth Affiliated Hospital, Sun Yat-sen University, Guangzhou, 510655 China; 2 Putuo People’s Hospital, Department of Bioinformatics, Tongji University, Shanghai, 200092 China

**Keywords:** COVID-19, SARS-CoV-2, Fever, Febrile, Critical

To the Editor:

Recent cohort studies frequently reported low rate of fever in coronavirus disease 2019 (COVID-19) patients [1, 2], which was in sharp contrast with previous studies that reported > 98% of patients presented fever on admission [3, 4]. Fever is a protective response for infections and an important criterion in the diagnosis of COVID-19. To understand the prevalence of fever in COVID-19 and its correlation with other symptoms and outcomes, we conducted a chart review of 252 hospitalized patients from 15 participating hospitals in Guangdong, Hubei, and Jiangxi provinces, China, from January 19 to March 6, 2020 (Table 1). Diagnosis of COVID-19 was based on positive severe acute respiratory syndrome-related coronavirus-2 (SARS-CoV-2) reverse transcription-PCR test. Our study was approved by the institutional review boards of the Sun Yat-sen University and the participating hospitals.

**Table 1:** Demographics and clinical characteristics of COVID-19 patients on admission

	Total (*n* = 252)	Febrile (*n* = 197)	Non-febrile (*n* = 55)	*P* value
Age, years				
≤ 29	26 (10%)	16 (8%)	10 (18%)	0.121
30–49	106 (42%)	85 (43%)	21 (38%)	
50–69	94 (37%)	73 (37%)	21 (38%)	
≥ 70	26 (10%)	23 (12%)	3 (5%)	
Sex				
Female	110 (44%)	82 (42%)	28 (51%)	0.231
Male	141 (56%)	114 (58%)	27 (49%)	
Temperature, °C				
< 37.3	55 (22%)	0	55 (100%)	**< 0.001**
37.3–38.0	104 (41%)	104 (53%)	0	
38.1–39	80 (32%)	80 (41%)	0	
39.1–41	13 (5%)	13 (7%)	0	
Signs and symptoms				
Cough	177 (70%)	145 (74%)	32 (58%)	**0.027**
Myalgia	42 (17%)	33 (17%)	9 (16%)	0.946
Cephalalgia	22 (9%)	12 (6%)	10 (18%)	**0.005**
Sputum	105 (42%)	92 (47%)	13 (24%)	**0.002**
Hemoptysis	4 (2%)	4 (2%)	0	0.579
Diarrhea	28 (11%)	23 (12%)	5 (9%)	0.590
Dyspnea	35 (14%)	28 (14%)	7 (13%)	0.768
Poor appetite	140 (56%)	117 (59%)	23 (42%)	**0.020**
Comorbidity				
Hypertension	48 (19%)	39 (20%)	9 (16%)	0.566
Diabetes	18 (7%)	14 (7%)	4 (7%)	1.000
Digestive tract disease	4 (2%)	2 (1%)	2 (4%)	0.444
Cardiovascular disease	10 (4%)	8 (4%)	2 (4%)	1.000
Cerebrovascular disease	3 (1%)	3 (2%)	0	1.000
Malignancy	4 (2%)	2 (1%)	2 (4%)	0.444
Liver disease	6 (2%)	3 (2%)	3 (5%)	0.234
Chronic lung disease	8 (3%)	5 (3%)	3 (5%)	0.512
Treatments and outcomes				
Oxygen supplementation	200 (79%)	157 (80%)	43 (78%)	0.806
Mechanical ventilation	10 (4%)	9 (5%)	1 (2%)	0.594
ECMO	4 (2%)	4 (2%)	0	0.579
Critically ill	52 (21%)	40 (20%)	12 (22%)	0.806
ARDS	21 (8%)	17 (9%)	4 (7%)	0.963
ICU admission	43 (17%)	34 (17%)	9 (16%)	0.876
Mortality	6 (2%)	6 (3%)	0	0.344

Data are median (IQR) or *n* (%). *P* values comparing febrile and non-febrile are from Mann-Whitney U test, *χ*^2^ test, or Fisher’s exact test, as appropriate

Medical records of COVID-19 patients were accessed from Jingzhou Hospital of Traditional Chinese Medicine (61 cases), Jianli Hospital of Traditional Chinese Medicine (41 cases), Jingzhou Central Hospital (21 cases), Dongguan People’s Hospital (14 cases), Jieyang People’s Hospital (8 cases), Shangrao People’s Hospital (12 cases), Shangrao No.2 People’s Hospital (3 cases), Poyang People’s Hospital (53 cases), Yugan People’s Hospital (3 cases), Wuyuan People’s Hospital (5 cases), Dexing People’s Hospital (3 cases), Guangfeng People’s Hospital (16 cases), Yushan People’s Hospital (9 cases), Yanshan People’s Hospital (2 cases), and Wannian People’s Hospital (1 case)

*ECMO* extracorporeal membrane oxygenation, *ARDS* acute respiratory distress syndrome, *ICU* intensive care unit

Demographic, clinical, laboratory, treatment, and outcome data were collected. The hospital course was reviewed for severity of disease. Critically ill patients were defined as those admitted to the ICU requiring mechanical ventilation or had a fraction of inspired oxygen (FiO_2_) of at least 60% [5]. SPSS (Statistical Package for the Social Sciences) version 24.0 software (SPSS Inc.) was used for Mann-Whitney *U*, chi-square, and the Fisher’s exact test. All statistical tests were two sided, with *p* values of < 0.05 considered to be statistically significant.

We found that, on admission, 197 (78%) patients had temperatures ≥ 37.3 °C, 93 (37%) patients had temperatures > 38 °C, and 13 (5%) patients had temperatures > 39 °C (Table 1). We then examined the differential symptoms and outcomes between febrile (≥ 37.3 °C) and non-febrile (< 37.3 °C) patients.

The most common symptoms on admission in both febrile and non-febrile patients were cough, poor appetite, and sputum production (Table 1). Smaller proportion of the non-febrile patients presented cough, poor appetite, and sputum production, compared to the febrile patients. In contrast, larger proportion of the non-febrile patients presented cephalalgia. The negative correlation between cephalalgia and fever is intriguing. Currently, there is no evidence for SARS-CoV-2 infection in brain tissue, although ACE2 expression in neuron was observed. Perhaps inflammatory cytokines from peripheral blood caused headache [6] in COVID-19. Cephalalgia may be a useful sign for the identification of non-febrile COVID-19, when epidemiological evidence for the infection exists.

No significant difference in any of the recorded comorbidities was observed between febrile and non-febrile patients.

Similar high proportions of febrile (157 [80%]) and non-febrile patients (43 [78%]) required oxygen supplementation (Table 1), indicating that respiratory system was the most affected system for both groups of patients. Among these, 17 (9%) febrile and 4 (7%) non-febrile patients developed acute respiratory distress syndrome (ARDS). Forty-three (17%) febrile and 9 (16%) non-febrile patients were admitted to the ICU. Critical illness was similarly common in the febrile (40 patients [20%]) and non-febrile (12 patients [22%]) patients.

Fever promotes inflammatory reaction, which may help control viral infection leading to beneficial outcomes. However, fever-induced upregulation of the inflammatory cytokines such as IL-1, TNF, and IL-6 may contribute to cytokine storm that contributes to critical illness. These double-edged effects of fever may explain the absence of correlation between fever and the disease outcome.

Given the high proportion of non-febrile patients in COVID-19, and that 20% non-febrile patients became critically ill, heightened attention for this elusive group of patients may be required for a better containment of the pandemic. Our finding is a timely alarm for health care workers and general population that temperature monitoring alone does not identify many of the COVID-19 patients.

## Data Availability

The data that support the findings of this study are available from the corresponding author upon reasonable request.

## Abbreviations

ARDS: Acute respiratory distress syndrome
COVID-19: Coronavirus disease 2019
FiO_2_: Fraction of inspired oxygen
SARS-CoV-2: Severe acute respiratory syndrome-related coronavirus-2

## References

[1] Goyal P, Choi JJ, Pinheiro LC, Schenck EJ, Chen R, Jabri A, Satlin MJ, Campion TR Jr, Nahid M, Ringel JB, et al. Clinical characteristics of COVID-19 in New York City. N Engl J Med. 2020. 10.1056/NEJMc2010419. 10.1056/NEJMc2010419 PMC7182018 32302078

[2] Richardson S, Hirsch JS, Narasimhan M, Crawford JM, McGinn T, Davidson KW, the Northwell C-RC, Barnaby DP, Becker LB, Chelico JD, et al. Presenting characteristics, comorbidities, and outcomes among 5700 patients hospitalized with COVID-19 in the New York City area. JAMA. 2020;323(20):2052-9. 10.1001/jama.2020.6775 PMC7177629 32320003

[3] Wang D, Hu B, Hu C, Zhu F, Liu X, Zhang J, Wang B, Xiang H, Cheng Z, Xiong Y, et al. Clinical characteristics of 138 hospitalized patients with 2019 novel coronavirus-infected pneumonia in Wuhan, China. JAMA. 2020;323(11):1061-9. 10.1001/jama.2020.1585 PMC7042881 32031570

[4] Huang C, Wang Y, Li X, Ren L, Zhao J, Hu Y, Zhang L, Fan G, Xu J, Gu X, et al. Clinical features of patients infected with 2019 novel coronavirus in Wuhan, China. Lancet. 2020;395(10223):497-506. 10.1016/S0140-6736(20)30183-5 PMC7159299 31986264

[5] Yang X, Yu Y, Xu J, Shu H, Xia J, Liu H, Wu Y, Zhang L, Yu Z, Fang M, et al. Clinical course and outcomes of critically ill patients with SARS-CoV-2 pneumonia in Wuhan, China: a single-centered, retrospective, observational study. Lancet Respir Med. 2020;8(5):475-81. 10.1016/S2213-2600(20)30079-5 PMC7102538 32105632

[6] Zhang X, Burstein R, Levy D. Local action of the proinflammatory cytokines IL-1beta and IL-6 on intracranial meningeal nociceptors. Cephalalgia. 2012;32(1):66-72. 10.1177/0333102411430848 PMC3674888 22144718

